# A System of Systems of Mental Health in Cities, Digging Deep into the Origins of Complexity

**DOI:** 10.1007/s10488-020-01035-0

**Published:** 2020-03-28

**Authors:** Elhabib Moustaid, Maksims Kornevs, Fredrik Lindencrona, Sebastiaan Meijer

**Affiliations:** 1 Royal Institute of Technology KTH, Hälsovägen 11, 141 52 Huddinge, Sweden; 2 Sveriges Kommuner Och Landsting, Hornsgatan 20, 118 20 Stockholm, Sweden

**Keywords:** Mental health, System dynamics modeling, Participatory methods, Urban health

## Abstract

Mental health in urban environments is often treated from a healthcare provision perspective. Research in recent decades showed that mental illness in cities is a result of dysfunctional coordination between different city systems and structures. Given the nature of the city as a system of systems, this work builds through a participatory method, a general system dynamic model of factors that affect mental health in urban and regional environments. Through this method, we investigated the challenges of the application of such methodology to identify essential factors, feedback loops, and dependencies between systems to move forward in planning for mental health in cities. The outcome is a general model that showed the importance of factors that vary from individuals, families to communities and feedback loops that span multiple systems such as the city physical infrastructures, social environments, schools, labor market, and healthcare provision.

## Introduction

Complexity science and systems thinking have gained more relevance in recent decades in many domains of application, including health care policy and urban planning. Complexity science has provided new ways of looking at cities providing tools to tackle wicked problems without neglecting their often pluralistic, interdisciplinary, multi-faceted nature (Batty 2013; Portugali 2012). Models and simulations provide a toolbox of investigation of the complexity (Batty and Torrens 2005). The late decades saw an increase in the use of models and simulations due to increasing modeling abilities and higher computational powers attributed to technological advances. These advances made modeling and simulation available outside the realm of scientific use, resulting in an expansion of the application of those methodologies to real-world, day-to-day problems. In healthcare notably, there is an embrace of systems thinking and modeling in different levels of applications; either on a macroscopic level of dealing with epidemics of disease or at a microscopic level of deciding the best treatments for patients (Bures et al. 2014). Carey et al. (2015) summarize particularly decades of application of systems and modeling approaches to public health policy. Their summary of 135 articles showed four main domains of application of system thinking in public health. First, studies that showed the potential of using such methodologies to investigate healthcare. Second, instances that used system thinking concepts to analyze public health data. Third, studies that benchmarked best practices through system methodologies, and finally, approaches that aimed at duplicating real systems.

This article tackles the issues of modeling mental health in cities and urban regions. According to World Health Organization (2004) mental health is a ‘state of complete physical, mental and social well-being and not merely the absence of disease or infirmity’. Reaching such a state proves to be challenging especially in urban environments. We investigate the application of a participatory model building approach to constructing a System dynamic (SD) model of city mental health. The goal is to investigate the city-systems concerning mental health, lay the groundwork to identify the significant causal loops in such a system, and the bridges that exist between these systems when it comes to reaching mental health objectives. The model building has taken place during the International initiative for mental health leadership (IIMHL) Conference in Stockholm 2018. The workshop elicited the knowledge of a group of stakeholders and experts with multiple backgrounds to build a general model of city mental health. It also explored their attitude and acceptance of such a process and its results.

The remainder of this paper is as follows. The next section presents the background of this work. The third section exhibits the participatory model building journey, including the discussions that took place during the process. The fourth section presents quantitative analysis and results. The fifth section discusses the outcomes, and then the conclusions are shared in the last section.

## The Complexity of Mental Health in Cities

With high rates of urbanization [55% of World Population lives in cities compared to 34% in 2000 (World Health Organization 2014)], cities find themselves constantly challenged to provide their populations with the best living conditions. Urban environments are attracting more people for all the opportunities they provide in terms of education, employment, and healthcare provision. The growth of cities, in term of populations, services and physical environments have led today to issues that manifest themselves in the level of the health of city populations (World Health Organization 2017). While cities in underdeveloped and developing countries are seeking to make sanitation and waste disposal systems efficient to avoid the spread of infectious diseases, cities in the developed world are setting strategies to deal with an epidemic of chronic diseases. Some of these diseases are dependent on the city lifestyle (Lederbogen et al. 2011; Word Health Organization and UN Habitat 2016). Studies have shown mental health to be often degrading in cities compared to rural areas (Gruebner et al. 2017). Mental health in urban environments is consequently one of the most significant burdens on healthcare today. According to the World Health Organization (WHO), 20% of life-quality disabilities are attributed mainly to neuropsychiatric disorders, which, to be put in order of magnitude, are only second to cardiovascular diseases (22%) (World Health Organization 2018).

Social environments, physical and psychological safety or even housing and built environment affect the mental health of people living in cities (Evans 2003; Evans et al. 2003; Nagai et al. 2007; Warr et al. 1988). The study of urban mental health is shifting from a view where mental health planning and strategy is one that is the responsibility of healthcare provision systems to a problem where different systems covering different domains intersect and interact. Consequently, achieving a city with an excellent mental health status is a wicked problem that needs the exploration of the city structures. Physical infrastructure, governance systems, organizational structures, civil society, family and social systems are examples of such structures and systems that need a careful investigation to bring answers to today’s challenges in mental health.

### International Initiative for Mental Health Leadership

The International Initiative for Mental Health Leadership is an initiative that seeks to tackle problems of mental health by empowering leaders in various ways. The initiative consists of a network of leaders from different cities around the globe. The events and collaborations enabled through the network are occasions in which exchange of expertise, experiences, showcases brings about valuable opportunities to develop, assess and discover ways of dealing with mental health issues. I-Circle (International and City and Urban Regional Collaborative) as part of the IIMHL focuses on cities. I-Circle, by involving cities and regions of eight different countries, shares experiences and innovations that tackle issues related to mental health. Besides, the network works on specific initiatives to encourage collaborative problem-solving and experience sharing between different urban centers and cities. These initiatives include preventive strategies, responding capacities and serving the population in the best way possible.

### The Contribution of the Paper

Mental health in cities and urban areas depends on many public sector institutions such as healthcare, education, labor market, and other structures in cities such as family, neighborhoods, communities, and other social support groups. Mental health requires such systems working together as cities status are the result of interactions of these systems (Portugali 2012; Raghothama et al. 2017). Furthermore, as there is no shared agency over all those systems, it is often hard to identify propagating effects across these systems and ways to remedy them. It is similarly hard to identify areas that can draw synergies between different systems to improve mental health and wellbeing. The objective of this work is to embrace the multiplicity of systems and agencies and build a model that can exhibit the factors that affect mental health in the cities and the relationship between those factors.

### Limitations

- The model building relies on a geographical and professional diversification of the participants. While this potentially makes the model diverse, it is limited to the scope of the knowledge of the participants.
- The elicitation of expert knowledge through this method only focused on the elicitation of factors that affect mental health in cities and how those factors affect each other.
- Network analysis is used to analyse the model in terms of centrality of factors. Further exploration of the model and its relevance in coordinated policy making is subject to another paper (Moustaid et al. 2019).
- The factors that are included in the model can lack rigorous definitions and can leave a lot of room for interpretation. Taking the time to define each factor rigorously can be part of future exercises.
- The validation interviews were done with participants selected randomly.

## The Model Building Journey

### Participatory System Dynamic Model Building Virtues

System dynamic (SD) models are models used to explore the dynamics of complex systems. SD models allow to represent complex dependencies between system components and identify causality loops that can affect the system or subparts of it. While SD models are good at explaining system behaviors, they remain a partial representation of the system. The challenge of building an SD model is to exhibit sufficient and necessary details for the model to be useful. SD applications in urban planning and healthcare help producing insights in a wide range of problems from land-use to obesity epidemic studies (Bures et al. 2014).

A participatory SD model building is a way of including relevant stakeholders in the process of building an SD model. This approach involves the beneficiary of the model in the scientific method of the model building. Participatory model building methods have been used for decades to study complex systems such as land-use and forest management (Schmitt et al. 2010; Stave 2010). Being practical and often easily actionable, these approaches have gained popularity in the circles of decision and policy making. Rigid mathematical models of systems often aim at building models that are inherently robust with high requirements for quantitative validity. Participatory methods while ‘softer’ than rigid mathematical modeling present methods that embrace the wholeness of systems without focusing on formal validity. They also provide tools to look at systems from different perspectives, to understand aspects that are otherwise impossible to investigate through mathematical lenses. Such aspects include stakeholder values and organizational aspects that are hard or impossible to quantify. A participatory approach for building a general system dynamic model, used in this work, is one that embraces the rigidity of SD as a mathematical method of representing and simulating systems at an elementary level, with a softer method consisting in stakeholder participation in the building process of the model.

### Grouping of Participants in the Participatory Model Building

**Fig. 1:**
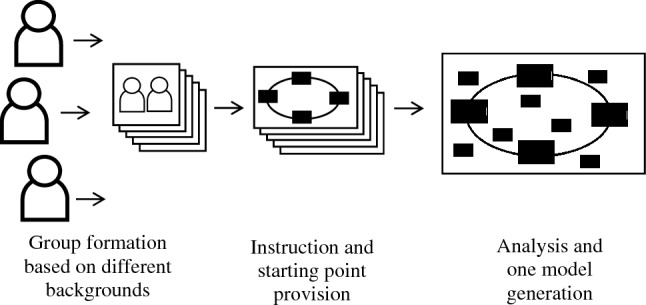
Participatory model building steps

Participatory modeling, as modeling in general, has no clear rules (Morgan and Morrison 1999). Stave (2010) shows different ways of participatory model building such as group and group size diversification or different methodologies used in workshops. The I-Circle workshop, during which this model building journey took place, had participants from different backgrounds both geographically and professionally. The model building was a process of eliciting their knowledge in the form of factors that can affect mental health and the nature of those effects. The model building journey formed groups with varied backgrounds and provided a starting point and the tools to build different models. After the analyses of the models, the models were merged into one general model. Figure 1 shows the steps of the model building journey.

The diversification of groups relied on two main axes. First, the groups were diversified to represent multiple geographic locations, hence composing groups with a diverse geographical backgrounds (Sweden, The United Kingdom, Canada, The United States of America, Spain, Australia). Second, the groups were diverse in terms of professional backgrounds; hence mixing individual coming from private sector with public sectors; as well as domain of profession; healthcare provision, academic research, politicians, law enforcement, or urban planners.

### Details of the Participatory Model Building

**Fig. 2:**
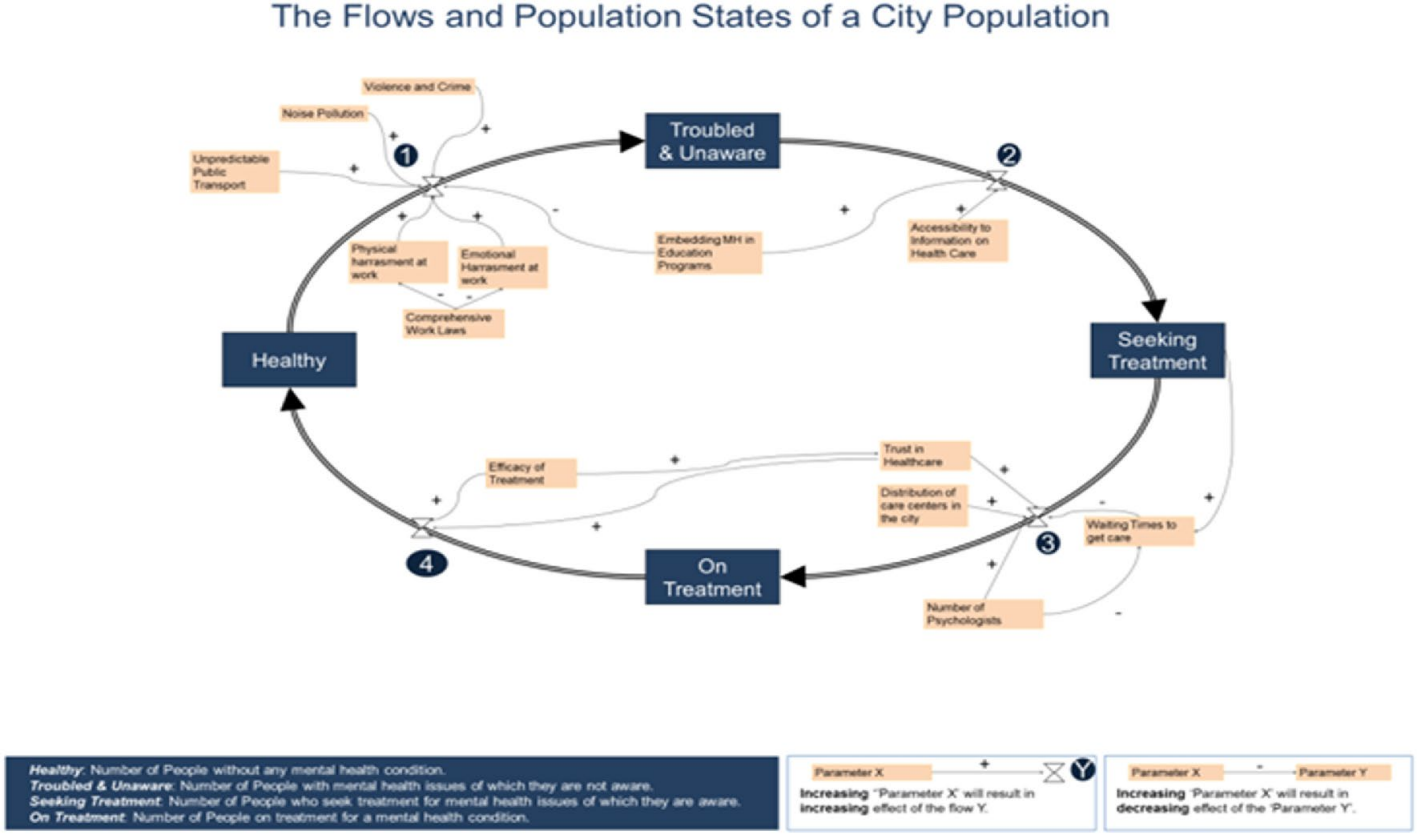
The starting model of the participatory model building exercise

With 40 participants, five diverse groups were built and were introduced to the SD methodology. Each group had the task of building a general model from a starting point. The starting model shown in Fig. 2 is an SD model that represented a city population as four stocks with four flows. The four ‘stocks’ are ‘states’ in which a person living in a city can be at a point in time, and a ‘flow’ is the number of people that move between those states by a time unit.

While the stocks were used merely to support the model building process, and hence, could be understood in different ways, the general definitions of the four stocks are the following,

- *Healthy*: the number of people living without any mental health challenge or their quality of life is not negatively affected by mental health issues at any level.
- *Troubled and unaware*: the number of people with any level of severity of mental health issues that affect their quality of life, but are not aware of the source.
- *Troubled and aware*: the number of people who seek treatment for any mental health issues of which they are aware.
- *On treatment*: the number of people that uses any type of treatment to cope with mental issues and to improve their mental wellbeing. Treatments for mental health issues could include but not limited by psychotherapy, medication, hospitalization, support groups.

Presenting mental health in such a flow model is already providing a platform to discuss general aspects of mental health planning. The model does not look at any specific issues nor have a specific agenda. However, as the model looks at urban mental as a complex whole, it does not intend focusing only on system subparts (such raising awareness and improving healthcare services). It instead intends to represent a larger picture of urban health. The flow 1 is a flow controlled by prevention as reducing that flow means preventing mental illness. The flow 2 is controlled by the level of awareness of the population. The flow 3 is one that represents the intervention capacity of the city. Finally, the flow 4 represents remission abilities of the cities, to move people from a recovery state to a healthy one. Hence the model is already leading the discussion in this direction. However, since those are some of the main discussions taking place today in mental health planning, and due to their generality as concepts, they represent a suitable start of the modeling journey (World Health Organization 2004).

The task of participants was then in a first step to find factors that can affect the flows between those states; this means factors that can decrease or increase the flow from a stock X to a stock Y. Participants were also asked to add factors that can affect any other factor already exposed in any way. The way a causal relationship is shown is through drawing directed lines with a sign ‘ + ’ or ‘ − ’. Hence a ‘ + ’ (respectively ‘ − ’) sign on a directed link from a factor ‘A’ to a factor ‘B’ means that increasing factor ‘A’ will result in increasing (respectively decreasing) effect on factor ‘B’. The sense of the direction is the sense of causality. In order to make the model is general, all voices need to be heard. Hence, every person in a group had the time to write down the factors that affect mental health in any capacity given their expertise. All individuals had 15 min to perform the task. After that, for 30 min, each participant in the groups had 20 s per factor in going around the clock.

**Fig. 3:**
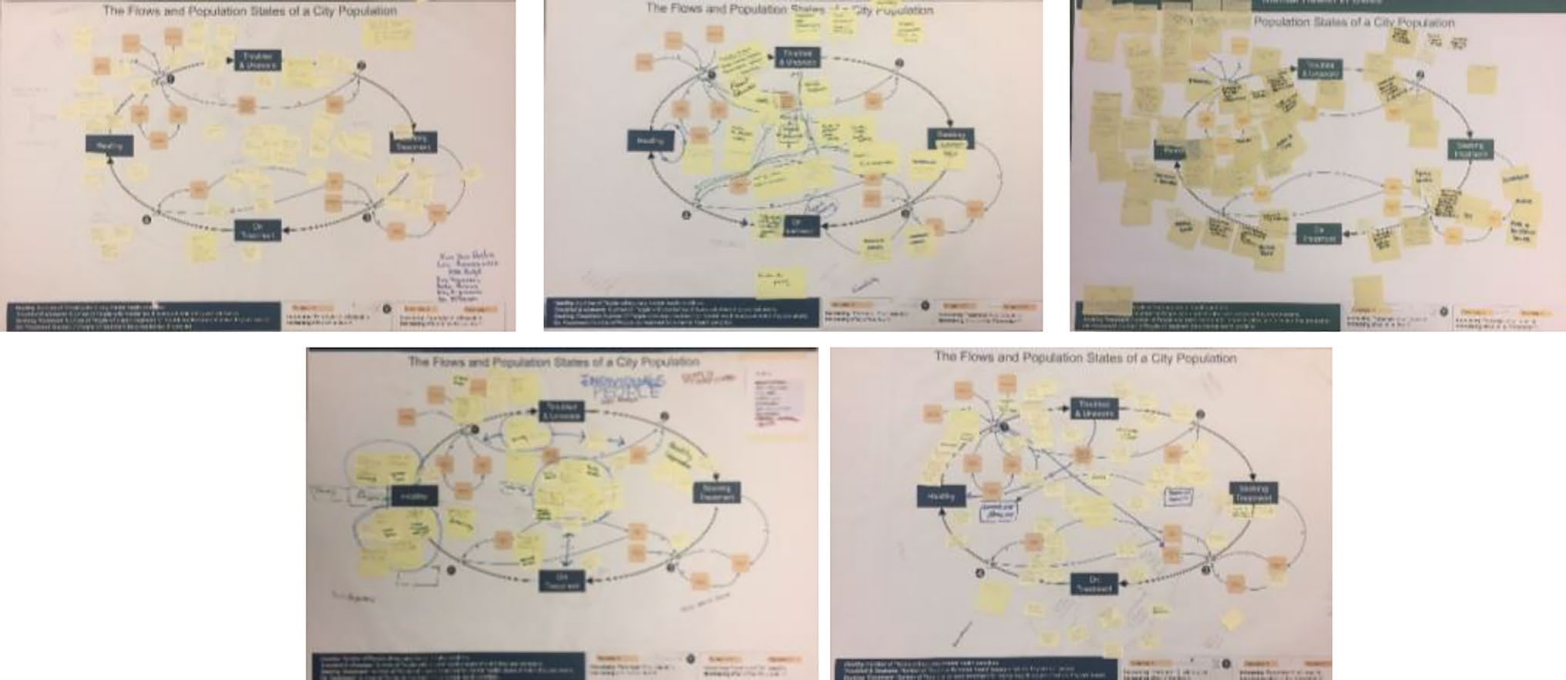
The outcome of the model building of the five groups involved

Figure 3 shows pictures of the outcome models.

### Overlaps and Differences in the Models

Five teams have delivered five different models. The models presented some differences and similarities. All teams agreed that mental health in cities requires coordination between different areas, groups, and agencies to be successful. Also, teams had a great focus on staying healthy and preventing issues with mental health, rather than fixing them later. Team A focused a lot on available resources and clarity on the rules how these resources are used in the context of mental health policy. Team B has connected all flows together because it is not just one big cycle and flows themselves can affect each other. Team C focused mainly on a connection between ‘healthy’, and ‘troubled and unaware’ in order to determine what contributes to this connection. They also addressed some issues in healthcare that are directly affecting mental health. Team D focused on a categorization of mental health problems and built their model around those categories. Team E added new stocks to measure aspects related to mental health generation.

During the model construction, participants have provided feedback on the model’s objectives, form, and language. First, on objectives of the model; few participants viewed the model to be focusing on an absence of disease perspective, and that mental health is not only a matter of health but also a matter of thriving and happiness. The ‘thriving’ dimension was lacking to the initial model and bringing it to the model was important.

Second, on the form of the model; some participants believed an important stock was missing, that stock was defined as self-care. The self-care refers to a non-neglectable portion of the population that has a mental health challenge but chose to deal with it through ways that do not include going through a traditional healthcare setting. From a modeling perspective, however, the self-care population is not entirely off the scope of the original model, as one can define self-care as a subpart of care, and hence is part of the stock ‘on treatment’. The fact that many groups came up with that stock means that it is a dominant and highly-interesting population. That population must be emphasized by the modelers easily visualized on the model. The alternatives for such an emphasis could be by explicitly showing the stock, or directly mention that self-care is a subpart of care in general.

Third, on the language used to describe the model; It was suggested to use the terminology ‘Recovery’ as opposed to ‘On treatment’. As 'Recovery' is what mostly happens between the treatment seeking and being healthy again. Using the term recovery is also a suitable one to consider a permanent mental disease. Recovery refers to the receiving of a treatment for mental illness and disorders without a differentiation between the gravity of the illness or its nature. The participants interpretation and use of recovery is one that is consistent with the definition advanced by (Onken et al. 2007) where recovery is defined the reestablishment of one’s mental health and its aftereffects on one’s other aspects of life.

Another terminology that was modified was ‘seeking treatment’ that changed to ‘troubled and aware’. This was suggested as all other stocks described states, except for ‘seeking treatment’ that suggested an action rather than a state.

### Converging to a General Model

**Fig. 4:**
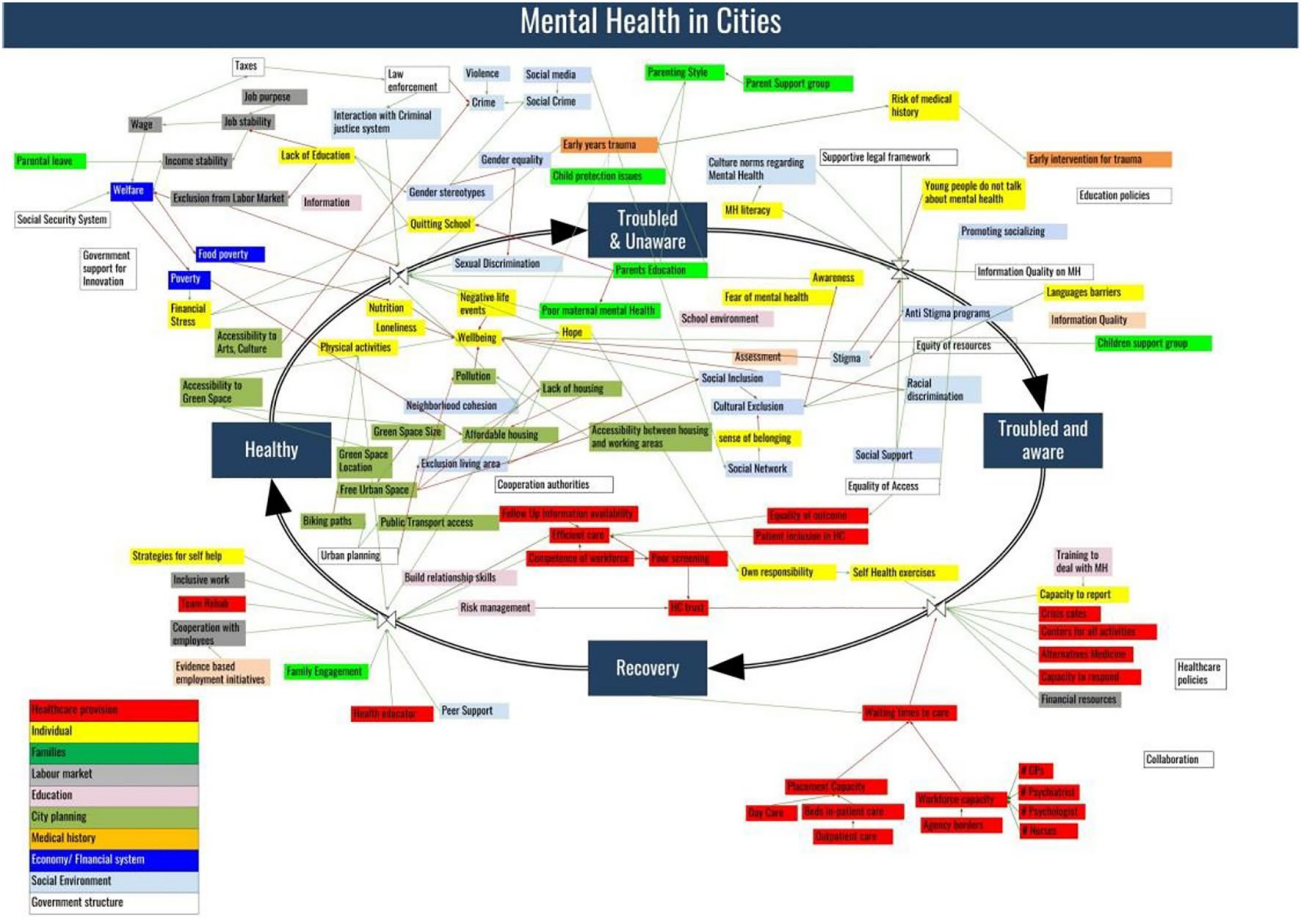
Model 1, A joint model constructed by joining the models in Fig. 3

In order to make sense of the data gathered, the modelers and moderators in a debrief session then reviewed the models and constructed a general model that takes elements from all models removing duplications and adding more links that can be substantiated with previous research or a follow-up interview with any of the participants. The modelers categorized the factors to systems to which they likely belong. Figure 4 shows the resulting model with we refer to from this point as Model 1.

A first debrief followed where the groups collectively assessed the outcome of the first model building session. Participants found the model to provide a good view at general problems regarding mental health in the city, but some aspects need to be considered as mental health is not only a health issue but also a thriving issue. The participants expressed interest in the potential use of the model for policy coordination for example. The system representation or coloring was judged as helpful for some participants as it helps to understand the areas of interactions between different systems and the level of those interactions.

The feedback on the model provided a new way of looking at the circle of health that was part of the original model. According to some participants, the flows were an easy way to show decision-makers across other sectors that prevention and remission, for example, are a joint responsibility of several systems together. This part is one that usually costs valuable resources in terms of direct expenses. Reducing those expenses can be reached possibly by looking at the right-top corner instead and reducing the flow of patients that go through healthcare through prevention strategies.

### A System of Systems View of the Model

In order to take previous remarks into account, a model that supports a different focal point was built from the same data. The same factors are kept but without a view of the cycle of care, i.e., ignoring the population status, in this presentation and focus instead on the elements of the system and their interactions constructing a cognitive map. A cognitive map is a graphical representation of a system relying on easy building blocks. They are often used to analyze causalities in system that are predominantly social (Tolman 1948; Kosko 1986).

The flows were altered and were represented, by the indicators associated with them:

- Prevention, an index of the efficiency of preventing mental illness. This is coupled with the Flow 1, Fig. 4. More prevention will decrease that flow.
- Awareness, an index of the level of awareness in the city, it is coupled to flow 2, Fig. 4.
- Intervention, an index of the capacity to intervene in the city. It is coupled to flow 3, Fig. 4.
- Remission, an index of the ability of recovery to be efficient and sufficient to regain a positive mental health status. It is coupled to flow 4, Fig. 4.

**Fig. 5:**
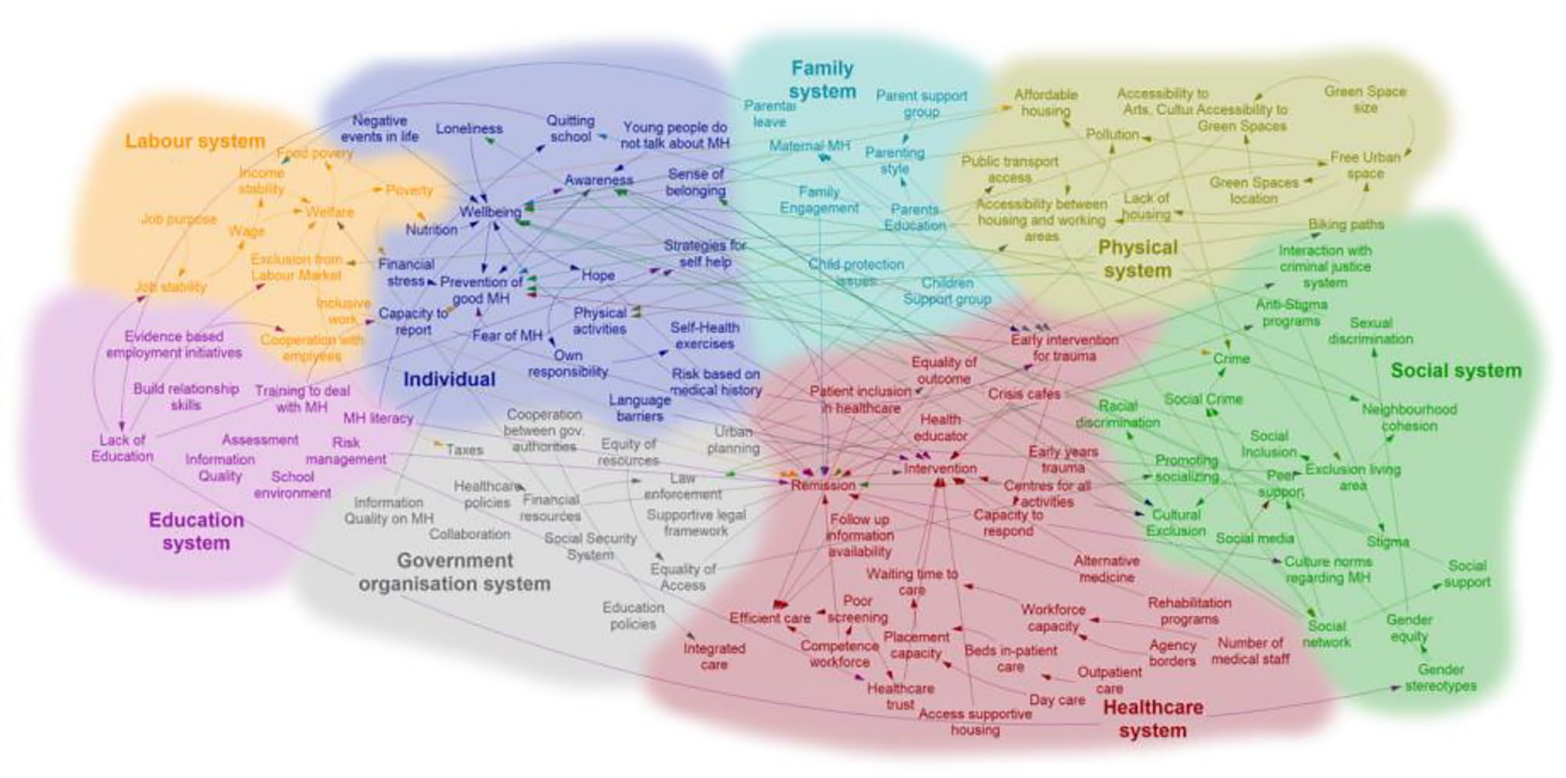
Model 2, a model that shows the Systems perspective in Model 1

The outcome of these changes is the model Fig. 5. The difference between Model 1 (Fig. 4) and Model 2 (Fig. 5) is solely on presentation. Model 2 allows seeing a system of the systems view of mental health in the city without linking it at first with a health status of the population. This new focal point allows seeing all the factors in their systems. It also allows for visualizing the interactions between the systems through the elementary interactions between their components.

### Qualitative Validation

Selected participants were interviewed in an open interview format about their perception of the model given their professional background and the city they were representing. The two questions that were asked were about their view of the model validity and if it would be a useful tool for them to plan mental health going forward. The model was deemed to be a valid representation of the outcome of the exercise that resulted in building it. The primary outcomes of those interviews were on the generality and completion of the model; i.e., the model captures major aspects of a city. The possible use of the model was still posing many questions. One major common hurdle to direct use in a city context was the specificity of the model to local city problems and governmental structure. For instance, a politician representing a tough neighborhood in a major European city expressed concern if this model does represent enough the picture from which originate the problems of their constituency. The model is a representation of a well-developed, safe city with no extreme violence or poverty. It can be challenging for the model to be useful for a city with tough neighborhoods according to that participant. The model does indeed contain poverty as a factor but using the model for the context of the politician might require more details than just that factor.

Specificity of governmental structure is also needed for the model to be useful. Such specification can include the determination of factors that belong to the responsibility of the local, regional, and national government.

The models were clear visualizations of the image of mental health in cities according to many participants. The model as it was presented could be a catalyzer for discussions around the role of city systems regarding mental health. The exploration of the model feedback loops and the analysis of each factor impacts can provide new ways of initiating policy coordination across different sectors, agencies, and actors in the city.

## Quantitative Analysis of the Final Model

The main model (Fig. 5) consists of 111 factors, linked with 180 links and belonging to 8 systems. The systems to which factors were clustered are Labor System, Education, and School System, Government Organizations, Family System, Healthcare, Physical Infrastructure, Social Systems, and Individual System.

### Feedback Loops

**Fig. 6:**
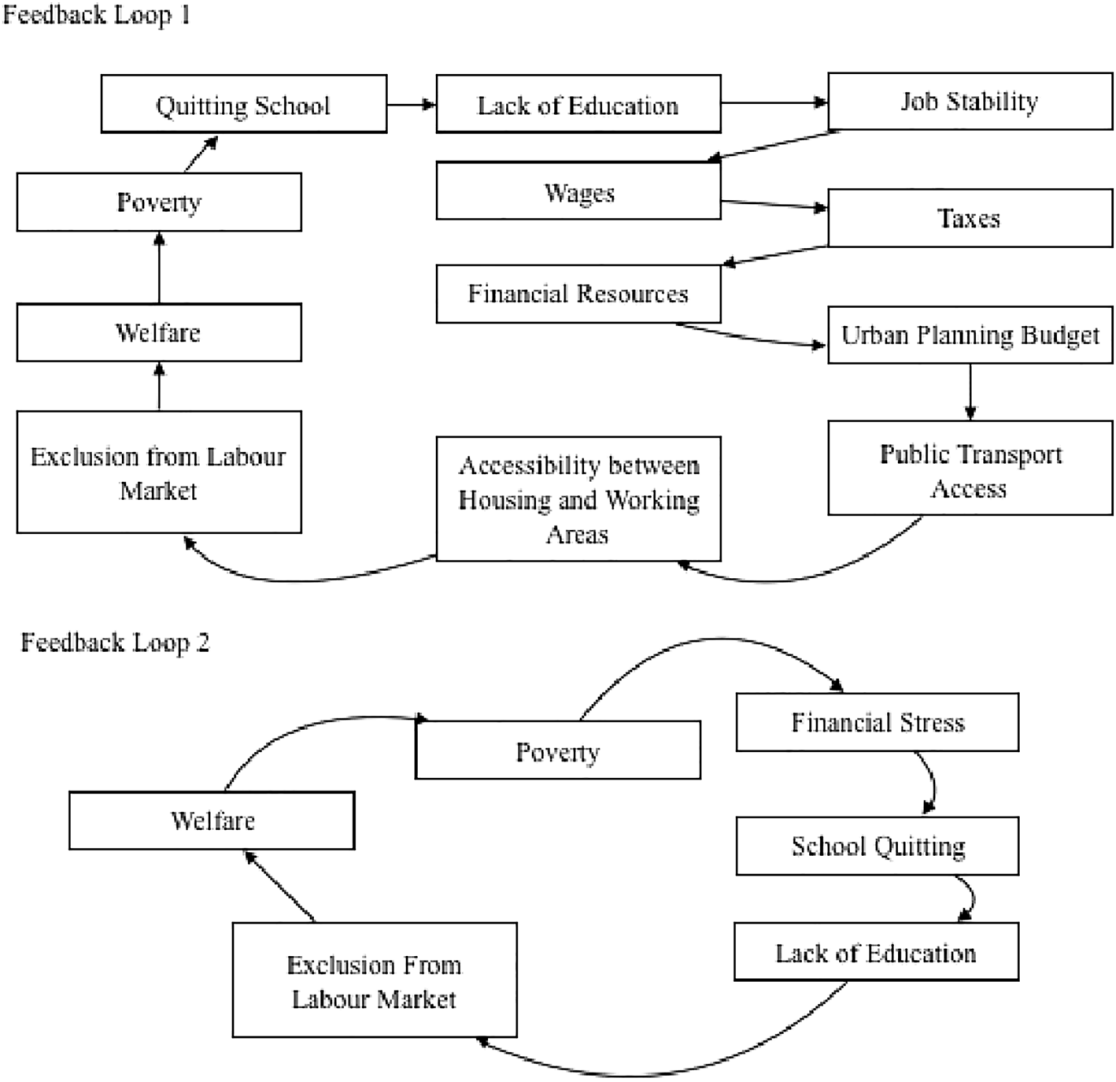
Examples of feedback loops

The main model contains about 535 feedback loops. Figure 6 shows two feedback loops that span over different systems. These two feedback loops are shown only as examples and not as the most important examples in the model. Both loops are balancing.

The first one shows a complex feedback loop with 12 factors across different systems. It shows that school dropouts can experience difficulties with job stability due to their lack of education. Such instability at the individual level can be consequent at a system level as it can reduce the financial resources in the city and its growth. City financial resources go to different areas depending on budgets set by local and regional or even national governments. One area that is concerned with such a budget is urban planning, which in turn can affect public transportation, and consequently accessibility of working areas to housing areas. If the accessibility is low, it will indeed contribute to excluding people from the labor market. That, in turn, could result in placing more people on welfare programs and impoverish them. This will make them live under financial stress, which in turn could result in other family members quitting school early to find sources of income. This closes the loop. This loop takes place over a long-term chronologically. It affects many systems at different levels along the way. This feedback loop relates directly to mental health status in the fact that job stability or access to public transport are sources of stress, which can result in severe mental illness or be a hurdle for remission plans.

The second example in Fig. 6 shows a shorter feedback loop. Financial stress is shown to increase school quitting, which in turn results in lack of education. The latter results in exclusion from the labor market, which in turn causes poverty, and hence generates financial stress again. The effect of this loop on mental health is similar to the one exposed in the previous example.

The effect of these feedback loops on the rest of the system can investigated through looking at the effects of the factors in these feedback loops on the rest of the system. For example ‘access to public transportation’ can affect mental health as its lack can decrease accessibility to work, which can result in financial stress in case of unemployment, hence increasing stress and anxiety. In the model, those factors affect the factor prevention which is the factor that determines how well is a city in keeping its inhabitants mentally healthy.

### Analysis of Model Factors and Components

This analysis takes advantage of the structure of the model in Fig. 5 as a directed graph a directed graph of the propogation of effects of factors affecting mental health in the city. It allows understanding the main model without the need for going through every factor and feedback loop. We extract the following results using the model as a network, where factors are the nodes, and the links are the edges with equal distances.

### Factor Reach Analysis

The factor that has the most reach in the model, i.e., the factors which effects can propagate the most through the model is the ‘*Financial resources’*. A factor ‘A’ is said to reach a factor ‘B’ if there is a path from that factor ‘A’ to the factor ‘B’ in the network.

‘*Financial resources’* is defined by the amount of financial resources that are available to a city through taxes, but also through other sources such as trade or labor. An effect on such factor can reach as many as 61 factors, i.e., 55% of all the factors in the model. This does not say anything however on the level of the influence or its nature. This result, however, is not a surprise as the city financial resources can be a determinant of the city development in many aspects. In a city management level, the budgets of cities can affect multiple systems at once. At the individual city inhabitant’s level, stable financial resources can usually be beneficial to the stability of individuals, families, and communities, as well as being part of the income to city districts and region (through taxation for example).

### Factor Centrality Analysis

To understand the position of nodes in the model we perform a centrality analysis that exhibit the centrality of the nodes in the graph. Closeness analysis allows to find how central are nodes as it counts a centrality score based on how distant is each node from the rest of the nodes in the graph. To perform this analysis, the distance associated to each link between two factors is set equal to 1. In the context of this model, this can allow to understand the ability of some factors (nodes) effects to reach other factors in the model.

**Fig. 7:**
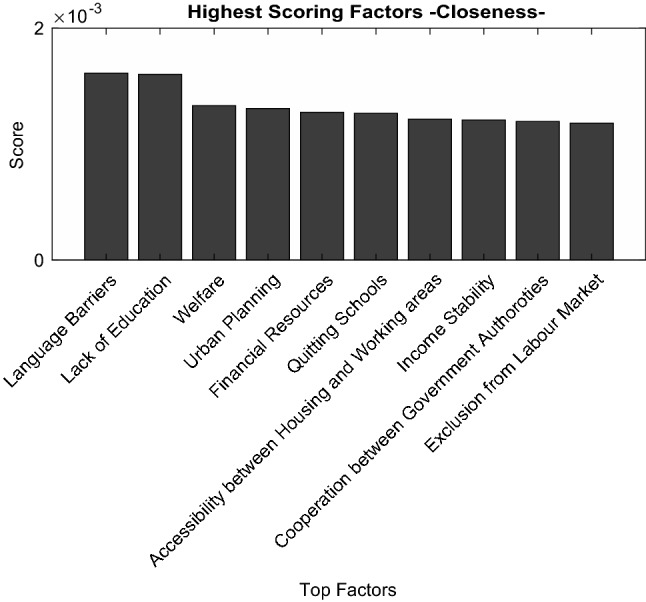
Top closeness scoring factors

Figure 7 exhibits the 10 most central factors in the model. The score computation is done by computing the distance of each factor to all other factors in the model. Hence the factor with the least distance to all other factors is one with the highest centrality. The most influential factors in the sense of centrality are *‘Language barriers’* and *‘Lack of education’,* followed by ‘*Welfare’*, ‘*Urban Planning’,* and ‘*Financial Resources’.* These factors belong to different systems showing one more how mental health is a multi-system issue.

Following the factors ‘*Language Barriers*’ and ‘*Lack of Education*’ effects show the importance of language and education. Language barriers are an obstacle for city inhabitants to get the information needed on healthcare, besides its effects on possible exclusion from the labor market, or effects on building social networks and a sense of belonging within the cities. Those are some reasons that can be part of increasing stress and anxiety that can lead people to disadvantageous mental health statuses. *‘Language barriers’* are also barriers to awareness, access to healthcare, or even having effective recovery plans*. ‘Lack of education’* as shown even in Fig. 5 can be influential in many ways, as it affects, job prospects, stability, or even awareness of mental health itself. This explains this factor has such a reach and influence in the model. Alongside those factors, ‘*Income stability’, ‘Accessibility between housing and working areas*’, and ‘*Quitting School*’ have relatively a high centrality.

## Discussion

The general model constructed through this participatory method shows the complexity of mental health in the city. The complexity shown in the model inherits the multitude of perspectives and thoughts of the stakeholders that built the model. The model is more than just a sum of its individual aspects or subgroups, and hence it is essential to address the issues and build a discussion around the complexity of the entire mental health system rather than just its individual elements. The outcome of the model building journey was a general model with two different focal points. It also resulted in discussions around the model validity, generality, specificity, completion, and usefulness. There are always means to improve, add and modify some of the model aspects.

The model represents a wide variety of opinions and backgrounds. It is a representation of truth that is partial due to the nature of its building. It has however produced consistent results when analyzed through various methods and produced feedback loops that can be analyzed and used to frame and build strategies. The model has, however, to be substantiated with more information to be specific to the domain of application. That means specifying the model to a specific city, agency structure, or adding or removing factors of interest or discard areas of the model to focus on specific areas only.

Specifying the model does not mean reducing its complexity, while simplification would mean reducing the model to a level where it becomes easily manipulated. Specifying it means to make it fitter to a context of use. An example is given from the conference by a government strategist who talked about the possible exploration of the prevention flow through enrichment of the model with local data and research to see how to come up with more prevention strategies. Another participant talked about using the model only if factors, systems agency and government levels were specific, meaning again there is a need to fit the model to a context and not to simplify it. Overall, this was a result of the participants' general attitude of wiliness to accept new methods that embrace complexity instead of operating in the realm of abstract simple conceptual models.

The participatory model building has been then successful at identifying factors of different systems, and their interactions in relation to mental health. However, there is an observation on the nature of those factors. Some of them are observable physical or social entities, while some are general or performance indicators that belong to systems. Example of social and physical entities is for example ‘*Green zones size*’, ‘*Pollution*’ or ‘*Health educators*’, and an example of an abstract notion is for example ‘*Strategies for self-help*’. While physical and social entities are easily measurable, defined, and quantifiable, abstract indicators and factors are often more complicated to measure or to represent in the model. This is one symptom of building the model with participants who generally operate at a strategic or managerial level where conceptual models are used.

The result of the graph analysis and feedback loops show the complexity of the problems faced in urban mental health today. Many feedback loops go across many systems, and the most influential factors are shown to influence and be affected by factors often belonging to different systems with different agencies. Financial resources, education (including mental health), language barriers, job stability having a big reach in the model and having each of them a responsibility that is multi-fold and not specific to one agency show that urban mental health can only be approached through system perspectives.

## Conclusion

The participatory mental health SD model building was a challenging process. The approach presented in this paper showed particularly the toughness of building a model that fits all but also succeeded to a large degree to characterize aspects that are relevant to stakeholders involved. The model being a general model is a way to help leaders working on mental health to consider a multitude of aspects that can affect them and which they would not account for otherwise. The model claims generality, but it is also naturally bound by the process it was built with, the participants who built it and their views, and the final modelers who finalized it. The generality of the model is best fitting the cities that participants came from, i.e., major cities and region of developed countries, and hence the model does not claim representation beyond cities and regions outside that category.

The results of the model and its analysis showed the validity of some aspects and revealed factors that are important regarding mental health including literacy of mental health, stable jobs, education, and city growth. It also shows that mental health efficiency is not only healthcare systems capacity to respond but is a significant problem that requires looking beyond systems boundaries. While healthcare provision was part of the model, it was shown to be only one of many systems which share effects and influences on mental health. Specifically, the model showed that proper management of resources and empowering people to take responsibility of their own lives and providing means to that, such as stable living conditions at work, home, in schools, in communities and cities is of utmost importance to reach best mental health results for the city populations.
